# Treatment Patterns, Health Care Resource Utilization, and Cost in Patients with Myelofibrosis in the United States

**DOI:** 10.1093/oncolo/oyab058

**Published:** 2022-02-04

**Authors:** Ronda Copher, Arianna Kee, Aaron Gerds

**Affiliations:** Bristol-Myers Squibb, Summit, NJ, USA; Bristol-Myers Squibb, Summit, NJ, USA; Department of Hematology and Medical Oncology, Cleveland Clinic, Cleveland, OH, USA

**Keywords:** myelofibrosis, ruxolitinib, burden, costs, health care resource utilization

## Abstract

**Background:**

This study analyses treatment patterns, health care resource utilization (HCRU), and costs in patients with myelofibrosis (MF) and a subgroup treated with ruxolitinib (RUX).

**Materials and Methods:**

Treatment patterns, all-cause and MF-related HCRU, and costs were analyzed in adults with MF with continuous enrollment in a commercial or the Medicare Advantage health plan in the pre-index period, defined as the 12 months immediately prior to the index date (date of primary or secondary MF diagnosis), and the post-index period, defined as ≥6 months following the index date. In a subgroup analysis, outcomes were analyzed in patients treated with optimal RUX (OPT RUX, ≥30 mg) and suboptimal RUX (SUB RUX, <30 mg) in the pre-index RUX period, defined as the 3 months immediately prior to the index RUX date (first date for an RUX claim), and the post-index RUX period, defined as ≥6 months following the index RUX date.

**Results:**

Of 2830 patients with an MF diagnosis, 1191 met eligibility requirements. The median age of patients was 72 years, 54% were male, and comorbidities were frequent. Sixty percent of patients received ≥1 line of therapy (LOT), of which 46% (*n* = 331) had ≥2 LOTs during the post-index MF period. Costs increased considerably 6-month pre-index to 6-month post-index (all-cause: cause ($24,216 to $48,966) and MF-related ($16,502 to $39,383), driven by inpatient stays and pharmacy costs. In the subgroup analysis, patients treated with RUX (*n* = 495) experienced significant disease burden and high costs, regardless of dose. A shorter duration of therapy and a higher rate of discontinuation were observed in patients treated with SUB RUX (*n* = 191) versus OPT RUX (*n* = 304).

**Conclusion:**

These findings suggest a significant disease and economic impacts associated with MF patients that persists with RUX therapy, highlighting the need for additional therapeutic options for MF.

## Teaser

Myelofibrosis leads to significant resource utilization and costs for patients and the US health care system. This article describes treatment patterns, health care resource utilization, and costs associated with myelofibrosis and in a subgroup of patients who were treated with ruxolitinib.

**Implications for Practice**

There is a lack of real-world evidence describing treatment patterns, health care resource utilization (HCRU), and costs among patients with myelofibrosis (MF). This retrospective study of 1191 patients with MF in the US revealed that treatment options are limited, and major drivers of costs include inpatient hospitalization and pharmacy. A subgroup analysis showed significant HCRU and costs remained in patients treated with ruxolitinib, regardless of dose. These findings highlight the substantial clinical and economic impacts associated with MF and the need for alternative treatments.

## Introduction

Myelofibrosis (MF) is a rare, chronic myeloproliferative neoplasm (MPN) characterized by bone marrow fibrosis, splenomegaly, cytopenias, and decreased survival.^1^ Other complications associated with MF include portal hypertension, thromboembolism, and frequent infections,^1^ and approximately 8-30% of patients with MF progress to blast-phase disease (acute myeloid leukemia).^2^ The estimated incidence of MF in the US is 1-3 new cases per 100 000 person-years.^3^

Myelofibrosis leads to significant health care resource utilization (HCRU) and costs for patients and the US health care system. Patients with MF have higher overall comorbidities, hospitalizations, and outpatient visits, which correspond to a 5-fold increase in health care costs compared with age- and gender-matched control patients without MPNs.^3^ Overall total utilization costs are higher among patients with MF compared with patients with other MPNs (ie, polycythemia vera and essential thrombocythemia).^3^

Treatment options for MF are limited and few patients undergo the only curative therapy, allogenic hematopoietic cell transplantation.^4^ The clinical manifestations of MF are heterogeneous and treatment choice is often complex, involving management of multiple symptoms including anemia, splenomegaly, constitutional symptoms, bone pain, and bleeding.^5^ Janus kinase (JAK) inhibitors, the only targeted therapies currently available for MPNs, were developed following the discovery of the *JAK2* V617F mutation as the most common recurring mutation in MPNs.^6,7^ From November 2011 to August 2019, the JAK1/JAK2 inhibitor ruxolitinib (RUX) was the only approved treatment for intermediate or high-risk MF, until the approval of a second JAK inhibitor fedratinib.^8^ Ruxolitinib improves splenomegaly and reduces symptoms in patients with intermediate-2 or high-risk MF.^9^ Analyses of long-term survival benefit with RUX based on data pooled from 2 phase III trials comparing RUX to placebo (COMFORT-I) or best available therapy (COMFORT-II) found that patients who received RUX had prolonged survival compared with patients treated with conventional therapies.^10,11^ However, JAK inhibitor therapy is associated with several complications.^12^ Some patients may develop dose-dependent RUX-related anemia and thrombocytopenia,^9^ which can lead to discontinuation. Dosing strategies may be used to mitigate anemia and thrombocytopenia; however, this may result in suboptimal clinical outcomes.^13^ Some patients with MF with an initial response to RUX develop drug resistance to RUX after 2-3 years of treatment, possibly due to only modest effects of RUX on driver mutation burden.^8^ There is no clear indication of a disease-modifying effect with RUX or other JAK inhibitors, with only a limited impact on induction of complete hematological remission and normalization of blood counts.^14-16^ These challenges and limitations highlight the need for alternative MF therapies.

Few real-world evidence studies have examined recent treatment patterns, HCRU, and cost among patients with MF and those treated with RUX.^17-20^ The objective of this study was to characterize the treatment patterns, HCRU, and costs associated with MF, and in a subgroup of patients with MF treated with RUX using a representative sample from the Optum Research Database (ORD).

## Materials and Methods

### Study Design and Data Source

This was a retrospective, observational study using administrative claims data from the ORD for adult commercial and Medicare Advantage health plan enrollees from January 1, 2011 to June 30, 2018. The ORD is one of the largest health care databases in the US containing de-identified medical and pharmacy claims data and cost information on more than 67 million beneficiaries from 1993 to present. In 2016, approximately 19% of the US commercially enrolled population, plus 17% of the Medicare Advantage, and 23% of the Medicare Prescription Drug Plan population were represented in the ORD.

### Patient Population

#### Primary analysis

The index MF date was defined as the first date for a primary or secondary MF diagnosis code during the identification period. Selected patients in the primary analysis met the following criteria: ≥2 non-diagnostic medical claims ≥30 days apart with diagnoses codes for primary MF (International Classification of Disease [ICD] code 238.76; D47.4) or secondary MF (ICD: 289.83; D75.81) in any position between January 1, 2012 and June 30, 2018; ≥18 years of age as of the index MF year; continuous enrollment in a health plan with medical and pharmacy benefits for 12 months immediately prior to the index date (pre-index MF period) and ≥6 months following the index date (post-index MF period); no evidence of pregnancy in the pre- or post-index MF periods; and no evidence of participation in clinical trials (ICD-9 dx: v70.7; Health care Common Procedure Coding System [HCPCS]: S9988, S9990-S9992, S9994, S9996) in the pre- or post-index MF periods.

#### Subgroup analysis

The index RUX date was defined as the first date for an RUX claim on or after the index MF date. Patients in the subgroup analysis met the first criterion of ≥2 primary or secondary MF non-diagnostic medical claims ≥30 days apart and additionally had ≥1 pharmacy claim for RUX on or after the index MF date, were ≥18 years of age as of the index MF year, had continuous enrollment for 3 months prior to the index RUX date (pre-index RUX period) and ≥6 months following the index RUX date (post-index RUX period), and had no evidence of pregnancy or clinical trial participation during the pre- or post-index RUX periods.

### Demographic and Clinical Characteristics

Age (index MF/RUX), sex, insurance type, and health plan regions were evaluated. Comorbidities were assessed in the 12-month pre-index MF period for the main analysis and in the 3-month pre-index RUX period for the subgroup analysis using Charlson Comorbidity Index (CCI) scores and Agency for Health care Resource Utilization (AHRQ)-defined comorbid conditions.

### Treatment Patterns

#### Primary analysis

Regimens received and duration of therapy through the first 2 lines of therapy (LOT) were examined. Treatment agents (ie, androgens, systemic steroids, erythropoiesis stimulating agents [ESAs], immunomodulatory imide drugs [IMIDs], iron chelation, and methotrexate) and procedures (ie, transfusions, splenectomy, splenic radiation, and transplantation) were captured in the 6-month pre-index and 6-month post-index MF periods.

An algorithm was used to identify the first 2 LOT in the main analysis. The first LOT start date was the date of the first National Comprehensive Cancer Network (NCCN)-recommended agent for MF or other systemic anti-cancer therapy claim in the February 2019 Chemotherapy List or transplantation or splenectomy after the index MF date. The first LOT end date occurred following the start of a new agent, discontinuation, transplantation or splenectomy, death, or disenrollment or study cut-off (June 30, 2018). First LOT regimens included all NCCN-recommended agents for MF and other systemic anti-cancer agents within the first 30 days of LOT start date. The most common regimens by LOT were reported. The second LOT start date was the first date of systemic anti-cancer therapy or transplantation or splenectomy claim after the first MF date after the first LOT end date.

#### Subgroup analysis

The following dosing patterns for RUX were assessed: RUX starting dose (dose on date of first RUX claim during the 6-month post-index RUX period), RUX starting dose duration, RUX max dose, RUX max dose duration, and discontinuation of RUX (defined as a gap of 45 days in the day’s supply for all RUX claims). Based on max dose, a suboptimal RUX (SUB RUX, <30 mg/day) cohort and an optimal RUX (OPT RUX, ≥30 mg day) cohort were defined.

Use of treatment agents and procedures were captured during the 3-month pre-index and 6-month post-index RUX periods.

### Health care Resource Utilization

All-cause and MF-related HCRU were calculated for ambulatory visits (office and outpatient), emergency department (ED) visits, and inpatient admissions, in the 6-month pre-index to 6-month post-index MF periods for the main analysis and during the 6-month post-index RUX periods in the subgroup analysis.

### Costs

All-cause and MF-related health care costs were computed as the combined health plan and patient paid amounts during the 6-month pre-index and fixed 6-month post-index MF periods for the main analysis, and 6-month post-index RUX periods in the subgroup analysis. Total costs were calculated as pharmacy and medical (including subcategories of ambulatory costs [office and hospital outpatient]) costs. Costs were adjusted to 2018 US dollars using the annual medical care component of the Consumer Price Index. Payments from Medicare and other payers were estimated based on coordination of benefits information obtained by the health plan.

### Study Measures and Analyses

All study variables were summarized using descriptive statistics. Frequencies and percentages were reported for categorical variables. Means, medians, and standard deviations (SD) were reported for continuous variables. *T*-test analyses were used for continuous variables and chi-square analyses were used for categorical variables. In the subgroup analysis, results were stratified by RUX dosing (SUB RUX: <30 mg/day and OPT RUX: ≥30 mg day).

## Results

### Primary Analysis

#### Patient attrition

**Figure 1.**
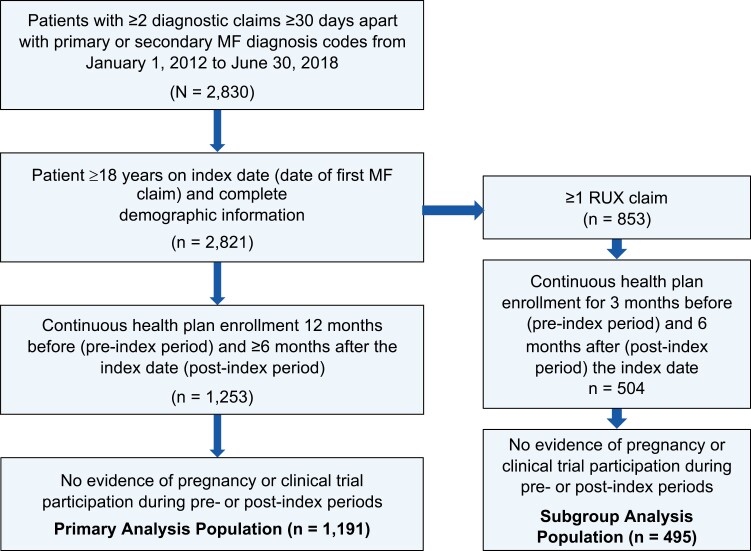
Patient attrition in the primary and subgroup analyses. MF = myelofibrosis; RUX = ruxolitinib.

Among 2830 patients with ≥2 medical claims with MF diagnoses codes, 1191 met the remaining selection criteria for the main analysis (Fig. 1).

#### Patient demographics and characteristics

Patient demographics and characteristics are presented in Table 1. The mean age of patients was 70 years. Fifty-four percent (*n* = 646) of patients were male and 24% (*n* = 291) had primary MF. The mean 12-month pre-index CCI score was 2.1, and 13% of patients (*n* = 155) had a CCI score ≥5. The 5 most common comorbidities during the 12-month pre-index period were anemia (71%), hypertension (66%), heart disease (57%), dyslipidemia (56%), and urinary tract disease (52%).

**Table 1.** Patient demographics and pre-index clinical characteristics among all patients with MF (primary analysis) and patients with MF treated with RUX (subgroup analysis).

Demographics/characteristics	Primary total *N* = 1,191	Subgroup total *N* = 495	SUB RUX *n* = 191	OPT RUX *n* = 304	*P-*value (SUB RUX vs OPT RUX)
Demographics					
Age, mean (SD)	70 (12)	69 (10)	71 (10)	69 (10)	.013
Male, *n* (%)	646 (54)	268 (54)	105 (56)	162 (53)	.631
Geographic region, *n* (%)					
Northeast	205 (17)	80 (16)	30 (16)	50 (16)	.828
Midwest	377 (32)	129 (26)	52 (27)	77 (25)	.640
South	451 (38)	213 (43)	90 (47)	123 (40)	.145
West	157 (13)	73 (15)	19 (10)	54 (18)	.017
Other	1 (0)	124 (25)	31 (16)	93 (31)	<.001
Pre-index clinical characteristics					
Charlson comorbidity index score, mean (SD)	2.1 (2.1)	1.5 (1.8)	1.6 (1.8)	1.5 (1.8)	.601
Most common AHRQ comorbidities, *n* (%)					
Neoplasms of unspecified or uncertain behavior	946 (79)	430 (87)	160 (84)	270 (89)	.106
Anemia	846 (71)	308 (62)	132 (69)	176 (58)	.009
Hypertension	788 (66)	233 (47)	100 (52)	133 (44)	.062
Heart disease	679 (57)	196 (40)	83 (43)	113 (37)	.164
Dyslipidemia	664 (56)	155 (31)	66 (35)	89 (29)	.218
Urinary system disease	619 (52)	185 (37)	74 (39)	111 (37)	.618
Other gastrointestinal disorders	602 (51)	254 (51)	100 (52)	154 (51)	.713
Other hematologic conditions	576 (48)	426 (86)	168 (88)	258 (95)	.334

AHRQ = Agency for Health Research and Quality; MF = myelofibrosis; OPT = optimal; RUX = ruxolitinib; SD = standard deviation; SUB = suboptimal.

#### Treatment patterns

Sixty percent (*n* = 718) of patients had at least one LOT, and among these patients, 46% (*n* = 331) (46%) had 2 or more LOTs during the 6-month post-index MF period. The top first-line (1L) and second-line (2L) regimens, duration of therapy, and reasons for ending treatment regimens are presented in Table 2. Ruxolitinib was the most commonly prescribed 1L therapy. Of 307 patients who were treated with RUX during 1L therapy (monotherapy or combination), 276 (91%) had at least 2 RUX pharmacy fills during 3 months of continuous enrollment after RUX initiation. Among these patients, 77 (28%) had an RUX dose modification during the first 3 months. In the 6-month post-index MF period, a variety of treatments were used (Supplementary Table S1); 37% of patients received a transfusion and 30% were treated with steroids.

**Table 2.** Summary of 1L and 2L therapy among patients with ≥1 line of therapy in the primary analysis (all patients with MF).

Top regimens and reason for ending	1L *N* = 718	2L *N* = 331
Top regimens, n (%)		
RUX monotherapy	281 (39)	90 (27)
RUX combination	26 (4)	26 (8)
Hydroxyurea monotherapy	226 (31)	94 (28)
Azacitidine or decitabine monotherapy	70 (10)	31 (9)
Other therapies	115 (16)	90 (27)
*Complete/uncensored LOT, n (%)*	500 (70)	234 (71)
Duration of complete lines, mean (SD) days	219 (281)	154 (228)
Reason regimen ended, n (%)		
Discontinuation (gap in therapy >45 days)	342 (48)	137 (41)
Switched to new medication	118 (16)	51 (15)
Transplantation or splenectomy	20 (3)	14 (4)
Death	20 (3)	12 (4)

1L = first line; 2L = second line; LOT = line of therapy; MF = myelofibrosis; RUX = ruxolitinib; SD = standard deviation.

#### Health care Resource Utilization

All-cause and MF-related HCRU increased from the 6-month pre-index to the 6-month post-index MF period (Supplementary Fig. S1A). All-cause and MF-related inpatient hospitalizations increased during the 6-month post-index MF period, from 261 (22%) pre-index to 360 (30%) post-index.

#### Costs

**Figure 2.**
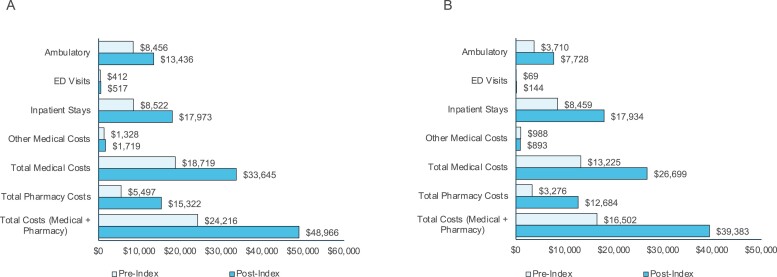
Mean 6-month pre-and post-index (A) all-cause and (B) MF-related cost of care at among patients with MF in the primary analysis. ED = emergency department; MF = myelofibrosis; OPT RUX = optimal ruxolitinib; SUB RUX = suboptimal ruxolitinib; TMC = total medical cost.

Mean all-cause and MF-related total medical costs were increased from the 6-month pre-index period to the 6-month post-index period (all-cause: $24,216 to $48,966 and MF-related: $16,502 to $39,383; Fig. 2). All-cause and MF-related costs were largely driven by inpatient hospitalizations.

### Subgroup Analysis

#### Patient attrition

In the subgroup analysis, 495 patients met the selection criteria (Fig. 1).

#### RUX treatment and cohort assignments

Median initial RUX dose was 30 mg/day and was continued for a mean (SD) of 70 (46) days. During the 6-month post-index RUX period, 19% of patients (*n* = 96) had at least one RUX dose modification. Median max RUX dose was 30 mg/day and was continued for a mean (SD) of 65 (47) days. The distribution of the maximum RUX daily dose was <15 mg: 68 (14%), 15-29 mg: 123 (25%), 30-40 mg: 276 (56%), and >40 mg: 28 (6%). Therefore, the SUB RUX cohort consisted of 191 patients, and the OPT RUX cohort contained 304 patients (Supplementary Fig. S2).

#### Patient demographics and characteristics

Patient demographics and characteristics in the subgroup analysis are presented in Table 1. The mean age of patients was significantly higher in the SUB RUX cohort than the OPT RUX cohort (71 vs 69 years, respectively, *P* = .013) and the cohorts did not differ significantly for 3-month pre-index comorbidities, except anemia (SUB RUX: 69% and OPT RUX: 58%, *P* = .009). Significantly fewer patients in the SUB RUX cohort had a primary MF diagnosis code than the OPT RUX cohort (16% vs 31%, *P* < .001).

#### Treatment patterns

**Figure 3.**
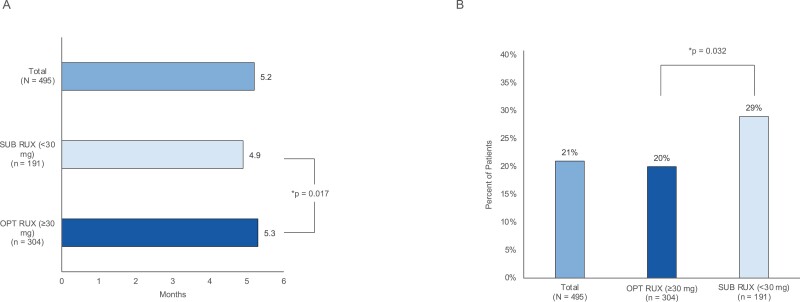
(A) Duration of therapy with RUX and (B) RUX treatment discontinuation in the subgroup analysis. OPT RUX = optimal ruxolitinib, SUB RUX = suboptimal ruxolitinib.

Over the 6-month post-index period, the average duration of RUX therapy was significantly shorter in the SUB RUX cohort compared with the OPT RUX cohort (4.9 vs 5.3 months, respectively, *P* = .017; Fig. 3A). A significantly greater proportion of patients in the SUB cohort discontinued RUX compared with the OPT cohort (29% vs 20%, respectively, *P* = .032; Fig. 3B). Nearly half of all patients in each cohort used a treatment agent during the 6-month post-index period (Supplementary Table S2). During the 6-month post-index period, a significantly higher proportion of patients experienced thrombocytopenia in the SUB RUX cohort compared to the OPT RUX cohort (31% vs 23%, respectively, *P* = .032) (Supplementary Table S2).

#### Health care Resource Utilization

Health care resource utilization was not significantly different among OPT RUX and SUB RUX cohorts for any outcome (Supplementary Fig. S1B).

#### Costs

Six-month mean post-index all-cause and MF-related costs are presented in Supplementary Fig. S3A and Fig. 3B, respectively. Medical costs trended higher for the SUB RUX compared with the OPT RUX cohort; however, the differences were not significant except for all-cause ED costs. All-cause ED costs were significantly greater among the SUB RUX cohort ($684) compared with the OPT cohort ($370) (*P* < .025). Statistically significant differences were demonstrated among cohorts for pharmacy costs. Six-month mean post-index all-cause and MF-related pharmacy costs were significantly greater among the OPT RUX cohort ($67,550 and $63,883) compared with the SUB RUX cohort ($57,265 and $54,282) (both values, < .001).

## Discussion

In this retrospective analysis, patients with MF had a high number of comorbidities, contributing to the considerable disease burden. Patients with MF also had a substantial economic impacts, as demonstrated by increased health care costs in the 6-month period following diagnosis. Primary cost drivers were inpatient hospitalization and pharmacy costs. The burden of MF was substantial, even with use of existing therapeutic options. Furthermore, a considerable proportion of patients remained untreated or experienced high rates of discontinuation after 1L therapy, and there were limited options for 2L therapy.

The findings from this study are aligned with a previous claims database analysis by Mehta et al^3^ In their study, patients with MF compared to age- and gender-matched control patients without MPNs had higher comorbidities (mean CCI: 2.1 vs 0.9), were hospitalized more often (34% vs 11%), experienced a higher number of average hospital days (7 days vs 1 day), and had more outpatient visits (58 vs 22). Similar to the current study, patients with MF incurred a higher average annual cost than matched comparisons ($54,168 vs $10,203), driven by medical ($45,646 vs $7,987) and pharmacy costs ($8,523 vs $2,216).

In the present study, patients with MF treated with RUX experienced high HCRU and medical costs, regardless of treatment dosage. Current NCCN guidelines recommend dosing of RUX based on platelet counts, as dictated by the US Food and Drug Administration (FDA) package insert, and indicate that specific clinical situations may support dose escalation strategies.^21^ In this study, 39% of patients received suboptimal RUX dosing, suggesting that many patients were not eligible to receive optimal dose levels. Due to limitations of claims data, patient prognosis was not evaluated; however, the lower RUX dosage may have been due to MF-related thrombocytopenia (platelet counts <100 × 10^9^/L) and MF-related anemia, both dose-limiting toxicities for RUX, and could have been indicative of more severe disease.^22-24^ Patients with platelet counts <50 × 10^9^/L were previously found to be most anemic and transfusion dependent, have the highest blast counts and an unfavorable karyotype, and short survival.^25-27^ The high frequency of anemia prior to treatment initiation among patients in the present study, including those receiving RUX, implies patients may have had more advanced disease, necessitating RUX dose titration.^28,29^ The HCRU and costs reported in the present study should be considered in the context of this high disease burden. Notably, all inpatient visits in the pre- and post-index periods appeared to be MF related. In addition, RUX treatment is associated with new-onset or worsening anemia and thrombocytopenia.^9,30^ Ruxolitinib dose-escalation regimens using a lower starting dose followed by incremental dose increases to lessen the severity and frequency of anemia and thrombocytopenia have been evaluated.^13^ In an open-label, phase II study, suboptimal clinical outcomes were associated with a lower RUX dose, and a clear dose–response relationship was demonstrated between RUX dosage and mean spleen volume, while mitigating worsening anemia.^13^ Spleen volume reductions at week 24 were greater with RUX >30-40 mg (32.9%) than RUX >20-30 mg (20.1%).^13^ Studies indicate that suboptimal dosing may have a negative impact on other clinical outcomes. In a previous open-label, phase II study by Talpaz et al,^13^ suboptimal clinical outcomes were associated with a lower RUX dose, and a clear dose–response relationship was demonstrated between RUX dosage and mean spleen volume.^8^ Spleen volume reductions at week 24 were greater with RUX >30-40 mg (32.9%) than RUX >20-30 mg (20.1%).^13^ Further studies are needed to understand the broader impact of suboptimal dosing on clinical effectiveness of RUX.

This study revealed that there is a high rate of treatment discontinuation and short duration of therapy associated with currently available MF therapies, including RUX. These patterns for RUX therapy were exacerbated by suboptimal dosing. Patients who received suboptimal RUX were shown to have significantly higher rates of discontinuation and significantly shorter duration of therapy compared with patients receiving optimal RUX dosage. A recent analysis by Palandri et al sheds light on the specific reasons for RUX discontinuation in patients with MF.^31^ In this study including clinical data for 524 patients from 20 European hematology centers, 51.1% patients had discontinued RUX therapy (mean treatment = 17.5 months), a majority of which experienced disease progression and lack of disease control. Of these individuals, reasons for discontinuation included death (18.7%), lack (22.9%), or loss (11.9%) of treatment response, RUX-related adverse events (27.5%), progression to blast phase (23.4%), adverse events not related to RUX (9.2%), and allogenic transplantation (5.1%).

Poor outcomes are reported among MF patients after discontinuation of RUX.^31,32^ For example, results from a US population-based outcome analysis showed that patients with MF had a median treatment progression-free survival of 6 months, and overall survival of 11 months, following RUX discontinuation.^32^ Risk of treatment progression or death was increased with age at discontinuation, CCI index score, and gender.

Treatment options are limited following RUX discontinuation or failure, which is higher among patients who are suboptimally dosed.^21^ The NCCN clinical practice guidelines now include fedratinib as a treatment option for intermediate-2 and high-risk MF, in patients who have previously failed or discontinued RUX.^21^ The addition of fedratinib expands the previously limited treatment options available for patients with MF.

### Study limitations

This observational analysis is retrospective in nature, as such, there are several limitations. First, the presence of a diagnosis code on a medical claim is not positive proof of the disease, as the diagnosis code may be incorrectly coded or included. Use of nondiagnostic medical claims for sample selection were applied to address this limitation. Second, the presence of a claim for a filled prescription does not indicate that the medication was consumed or taken as prescribed. Third, because study patients are enrolled in commercial or Medicare plans during the study period, the study findings may not be applicable to those patients who are uninsured or enrolled in other health plans. Fourth, missing information from the database may result in selection bias, confounding, or measurement error. In particular, patient MF risk stratification information, such as that captured using the International Prognostic Scoring System, Dynamic International Prognostic Scoring System (DIPSS), or the DIPSS-Plus, is not available in claims data and was not assessed in the present study. Similarly, data on high-risk mutations and karyotype pertinent to MF were not collected. Moreover, conclusions regarding the clinical efficacy of treatments for MF could not be made. Despite these limitations, claims data provide important insight into health outcomes in a real-world setting, which contains large samples sizes of patients with diverse medical histories.

## Conclusion

The results of this retrospective analysis demonstrate the substantial MF-related medical resource utilization and corresponding economic impacts in the US. This significant HCRU and costs remained in RUX-treated patients, regardless of dose. Patients who received suboptimal doses of RUX had a significantly shorter duration of therapy and significantly higher rate of discontinuation compared with patients who received optimal doses of RUX. This study highlights the current need for alternative treatments that provide options to patients who fail or discontinue treatment with RUX or other therapies and may offset the clinical and economic impacts of MF to patients, payers, and health care systems.

## Supplementary Material

oyab058_suppl_Supplementary_Figures

oyab058_suppl_Supplementary_Tables

## Data Availability

The data underlying this article will be shared on reasonable request to the corresponding author.
