# Supplementary material for: Treatment Patterns, Health Care Resource Utilization, and Cost in Patients with Myelofibrosis in the United States
Source: Oncologist. 2022 Feb 4;27(3):228–35. doi: 10.1093/oncolo/oyab058 (PMC8914486; doi:10.1093/oncolo/oyab058)

The Oncologist
The official journal of the Society for Translational Oncology

Supplemental Figures for:
Treatment Patterns, Healthcare Resource Utilization, and Cost in Patients with Myelofibrosis in the United States
Ronda Copher et al.

Supplemental Figure 1. Pre- and Post-Index HCRU Among A) Patients with MF in the Primary Analysis and B) Patients Treated with RUX in the Subgroup Analysis

Abbreviations: ED = emergency department, HCRU = healthcare resource utilization, MF = myelofibrosis, RUX = ruxolitinib

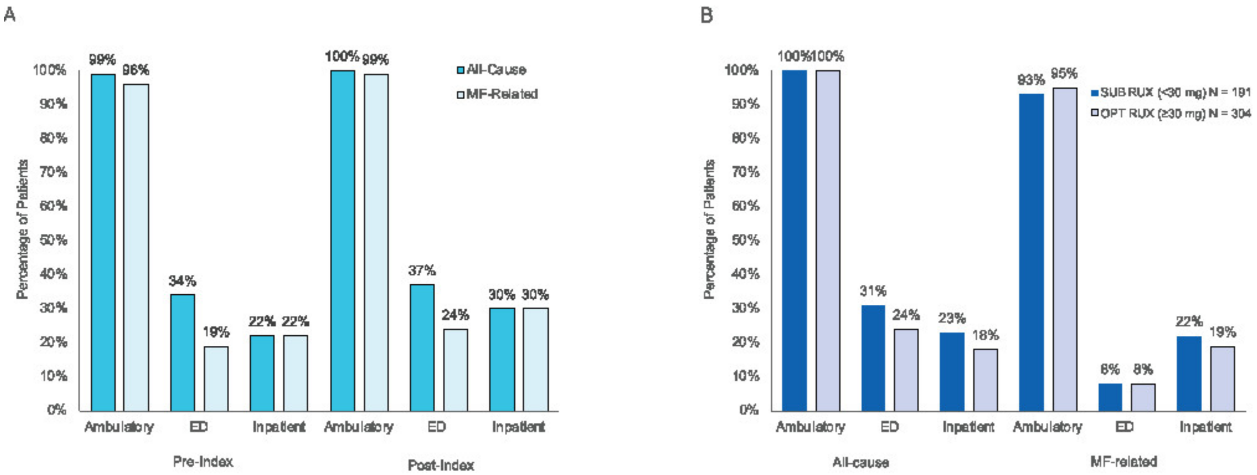

Supplemental Figure 2. RUX Treatment and Cohort Assignment in the Subgroup Analysis

Abbreviations: OPT RUX = optimal ruxolitinib, SUB RUX = suboptimal ruxolitinib

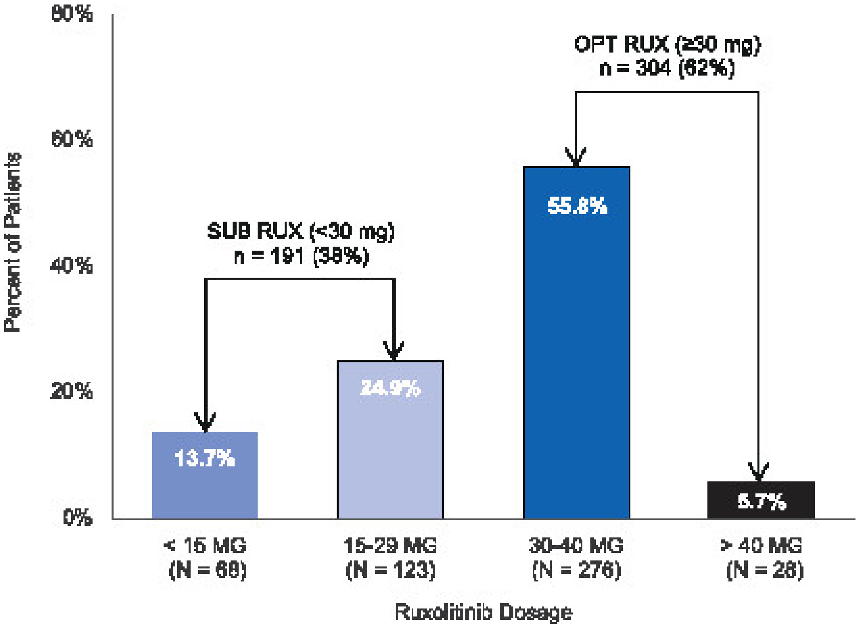

Supplemental Figure 3. Mean 6-Month Post-Index A) All-Cause and B) MF-Related Cost of Care Among Patients Treated with RUX in the Secondary Analysis

Abbreviations: ED = emergency department, OPT RUX = optimal ruxolitinib, SUB RUX = suboptimal ruxolitinib

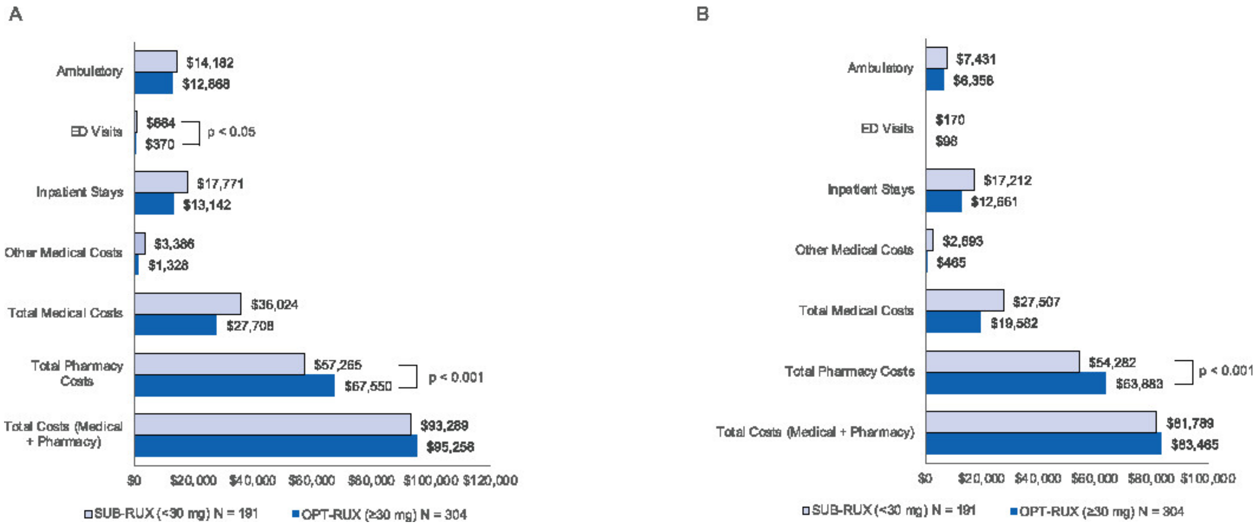

Supplement: oyab058_suppl_Supplementary_Figures [file oyab058_suppl_supplementary_figures.pdf]
