# Supplementary material for: Treatment Patterns, Health Care Resource Utilization, and Cost in Patients with Myelofibrosis in the United States
Source: Oncologist. 2022 Feb 4;27(3):228–35. doi: 10.1093/oncolo/oyab058 (PMC8914486; doi:10.1093/oncolo/oyab058)
Supplement: oyab058_suppl_Supplementary_Tables [file oyab058_suppl_supplementary_tables.pdf]

The Oncologist
The official journal of the Society for Translational Oncology

Supplemental Tables for:
Treatment Patterns, Healthcare Resource Utilization, and Cost in Patients with Myelofibrosis in the United States
Jan Schellens et al.

Supplemental Table 1. Summary of Supportive Treatments in the Primary Analysis (All Patients with Myelofibrosis)

**Supportive Treatment** (n, %)	**All Patients** N = 1,191
Transfusions	435 (37)
Systemic steroids	361 (30)
ESAs	187 (16)
Transplantation	33 (3)
Androgens	23 (2)
IMIDs	23 (2)
Thalidomide	15 (1)
Iron chelation	11 (1)
Methotrexate	8 (1)
Splenectomy	4 (0.3)
Splenic radiation	0 (0)

Abbreviations: ESA = erythropoiesis stimulating agent, IMID = immunomodulatory drug, MF = myelofibrosis

Supplemental Table 2. Post-Index Comorbidities and Treatment Patterns in Patients with MF Treated with RUX (Subgroup Analysis)

Comorbidities/Treatments	Total (N = 495)	SUB RUX (n = 191)	OPT RUX (n = 304)	*p* value
**Post-Index Comorbidities, n (%)**				
Anemia	324 (65)	131 (69)	193 (63)	0.245
Neutropenia	96 (19)	41 (21)	55 (18)	0.355
Thrombocytopenia	129 (26)	60 (31)	69 (23)	0.032
**Post-Index MF-related Treatments**				
Supportive Agents, n (%)				
Any supportive agent	225 (45)	92 (48)	133 (44)	0.337
Androgens	15 (3)	4 (2)	11 (4)	0.336
Systemic steroids	161 (33)	67 (35)	94 (31)	0.336
ESAs	92 (19)	39 (20)	53 (17)	0.406
IMIDs	4 (0.8)	2 (2)	2 (1)	0.638
Thalidomide	2 (0.4)	0 (0)	2 (1)	0261
Iron chelation	14 (3)	7 (4)	7 (2)	0.373
Methotrexate	1 (0.2)	1 (1)	0 (0)	0.207
Procedures, n (%)				
Transfusions	226 (46)	86 (45)	140 (46)	0.823
Splenectomy	1 (0.2)	0 (0)	1 (0.3)	0.428
Transplantation	9 (2)	4 (2)	5 (2)	0.716

Abbreviations: ESA = erythropoiesis stimulating agent, IMID = immunomodulatory drug, MF = myelofibrosis, OPT RUX = optimal ruxolitinib, SUB RUX = suboptimal ruxolitinib
